# Mitochondrial Control Region Alterations and Breast Cancer Risk: A Study in South Indian Population

**DOI:** 10.1371/journal.pone.0085363

**Published:** 2014-01-30

**Authors:** Nageswara Rao Tipirisetti, Suresh Govatati, Priyanka Pullari, Sravanthi Malempati, Murali Krishna Thupurani, Shyam Perugu, Praveen Guruvaiah, Lakshmi Rao K, Raghunadha Rao Digumarti, Varadacharyulu Nallanchakravarthula, Manjula Bhanoori, Vishnupriya Satti

**Affiliations:** 1 Department of Genetics, Osmania University, Hyderabad, India; 2 Department of Biochemistry, Osmania University, Hyderabad, India; 3 Department of Biotechnology, Periyar University, Salem, Tamilnadu, India; 4 Department of Biochemistry, DrMRAR PG Center, Krishna University, Nuzvid, India; 5 Department of Biotechnology, Chaitanya Postgraduate College, Hanamkonda, India; 6 Centre for Cellular and Molecular Biology (CCMB), Hyderabad, India; 7 Nizam’s Institute of Medical Sciences, Hyderabad, India; 8 Department of Biochemistry, Sri Krishnadevaraya University, Anantapur, India; Rajiv Gandhi Centre for Biotechnology, India

## Abstract

**Background:**

Mitochondrial displacement loop (D-loop) is the hot spot for mitochondrial DNA (mtDNA) alterations which influence the generation of cellular reactive oxygen species (ROS). Association of D-loop alterations with breast cancer has been reported in few ethnic groups; however none of the reports were documented from Indian subcontinent.

**Methodology:**

We screened the entire mitochondrial D-loop region (1124 bp) of breast cancer patients (n = 213) and controls (n = 207) of south Indian origin by PCR-sequencing analysis. Haplotype frequencies for significant loci, the standardized disequilibrium coefficient (D′) for pair-wise linkage disequilibrium (LD) were assessed by Haploview Software.

**Principal Findings:**

We identified 7 novel mutations and 170 reported polymorphisms in the D-loop region of patients and/or controls. Polymorphisms were predominantly located in hypervariable region I (60%) than in II (30%) of D-loop region. The frequencies of *310‘C’* insertion (*P* = 0.018), *T16189C* (*P* = 0.0019) variants and *310‘C’ins/16189C* (*P* = 0.00019) haplotype were significantly higher in cases than in controls. Furthermore, strong LD was observed between nucleotide position 310 and 16189 in controls (D′ = 0.49) as compared to patients (D′ = 0.14).

**Conclusions:**

Mitochondrial D-loop alterations may constitute inherent risk factors for breast cancer development. The analysis of genetic alterations in the D-loop region might help to identify patients at high risk for bad progression, thereby helping to refine therapeutic decisions in breast cancer.

## Introduction

Breast cancer is the most common non-cutaneous malignancy among females in the Western world and ranked second after lung cancer in mortality rates [1]. In India, it is the second most common cancer in females [2]. Genetic background, environmental exposures and gene-environment interactions contribute to the development of breast cancer [3]. Although epidemiologic investigations have identified numerous risk factors in the origin of breast cancer, the etiology and pathogenesis remain unclear [4]. Previously, we demonstrated the correlation between single nucleotide polymorphisms (SNPs) of various candidate genes and risk of breast cancer in Indian women [5]–[8]. The emerging evidence strongly suggests that the disease has polygenic and multifactorial basis [9]. Recent investigations have shown the potential involvement of reactive oxygen species (ROS) in breast carcinogenesis [10]–[12]. Mitochondria are a major source for ROS generation and mitochondrial DNA (mtDNA) alterations have been found in a variety of human diseases including cancer [13]–[17].

Mitochondria play an important role in energy metabolism, aging and apoptosis [18]. Human mtDNA is a 16.569 kb circular double-stranded DNA (dsDNA) molecule that encodes 13 polypeptide components of the electron transport chain (ETC), 22 tRNAs and 2 rRNAs [19]. mtDNA exhibits higher mutation rate than nuclear DNA and is more vulnerable to oxidative damage due to a lack of protective histone proteins, limited DNA repair mechanisms and a high rate of ROS generation [20].

The displacement loop (D-loop) is the only non-coding region [nucleotide position (np) 16024-576 = 1124 bp] of mitochondrial genome and is known to accumulate mutations at a higher frequency than other regions [21]. It is a hot spot for mtDNA alterations and comprises of two hypervariable regions (HVR1: np 16024–16383 and HVR2: np 57–333). The D-loop contains crucial elements for replication and transcription of mtDNA [22]. Hence, sequence alterations in D-loop region may contribute to altered replication and/or transcription of mitochondrial genes which may affect the overall mitochondrial function and cellular ROS generation. Accumulation of D-loop alterations has been reported in several complex human diseases [23]–[26] but studies related to breast cancer are very few [27]–[30]. Since no reports are available from Indian population, we conducted a case-control study to investigate the association between entire D-loop alterations and breast cancer risk.

## Materials and Methods

### Subjects and Sampling

Blood samples were collected from breast cancer patients (n = 213) and age, sex matched healthy controls (n = 207) of south Indian origin (Dravidian linguistic group) from Department of oncology, Nizam’s Institute of Medical Sciences (NIMS), Hyderabad, India. All the patients recruited in this study were medically confirmed primary breast cancer patients [diagnosed by mammotome biopsy and/or fine needle aspiration cytology test (FNAC test)] who had given written consent to participate in this study. The information on age at onset (pre menopausal = 73; post menopausal = 140), hormonal receptor status [estrogen receptor positive (Er +ve) = 98, negative (Er –ve) = 115); progesterone receptor positive (Pr +ve) = 104, negative (Pr –ve) = 109; human epidermal growth factor receptor 2 positive (Her2+ve) = 87, negative (Her2–ve) = 126], stage of the breast cancer [TNM system; stage I = 21, stage II = 80, advanced stage (stage IIIA, IIIB and IV) = 112], tumor size and linguistic background was documented through personal interview and also by verification of medical records. Peripheral blood samples (5 ml) were collected from all the subjects in EDTA vacutainers and stored at −80°C until further use. Ethical committee of department of Genetics, Osmania University, Hyderabad and Institutional Review Board of the Centre for Cellular and Molecular Biology (CCMB), Hyderabad, approved the study.

### DNA Extraction and Genotyping of Entire D-loop

Genomic DNA was extracted from peripheral blood samples following method described elsewhere [31]. Both cases and controls were genotyped in a randomized, blinded fashion. Genotyping of entire D-loop was carried out by PCR-Sequencing analysis as per the protocols described earlier [32]. PCRs were carried out in a total volume of 25 µl, containing 50 ng genomic DNA, 2–6 pmole of each primer, 1X Taq polymerase buffer (1.5 mM MgCl_2_) and 0.25 U of Amplitaq DNA polymerase (Perkin Elmer, USA). Two pairs of overlapping primers (Table 1) were used to amplify the entire 1122 bp D-loop region of mtDNA. The generated DNA fragments vary in size from 809 bp to 963 bp with an average size of 886 bp. The total size of amplified fragments was 1772 bp, 63.43% more than the whole D-loop because of the overlapping regions. The PCR products were sequenced with a Taq-Dye deoxy-terminator cycle sequencing kit (Applied BioSystems, Foster City, USA) using an automated ABI 3770 DNA sequencer (Applied BioSystems, Foster City, USA). Genotype calling was performed using Chromas V.2 software (Technelysium Ltd., Australia).

**Table 1:** Primers used in this study for entire D-loop sequencing.

Serial number	Primerset	Primer sequence 5′→3′	Nucleotideposition	Overlap(bp)	Ampliconsize (bp)	Tan^1^(°C)
1	1F	TCATTGGACAAGTAGCATCC	15792 - 31	–	809	58
	1R	GAGTGGTTAATAGGGTGATAG				
2	2F	CACCATCCTCCGTGAAATCA	16401 - 794	199	963	58
	2R	AGGCTAAGCGTTTTGAGCTG				

1 Annealing temperature.

### Mutational Analysis of Entire D-loop

The individual mtDNA sequences were compared against the Revised Cambridge Reference Sequence (rCRS) [33] using Auto Assembler-Ver 2.1 (Applied Biosystems, Foster City USA). Sequences were aligned using CLUSTAL X and mutations were noted by using MEGA software ver 3.1. Independent sequencing readings were performed by two different investigators (NRT and SG). Sequence variations found in both cases and controls were checked against the ‘mitomap database’. Those not recorded in the database were categorized as novel mutations, and those that appeared in the database were reported as polymorphisms.

### Statistical Analysis

Statistical analysis was performed using SPSS statistical package (V 11.0). The allele ratios and genotype distributions of cases and controls were analyzed using Fisher’s exact test. The odds ratio and 95% confidence interval (CI) values were calculated using the online Vassar Stats Calculator (http://www.faculty.vassar.edu/lowry/VassarStats.html). Haplotype frequencies for multiple loci and the standardized disequilibrium coefficient (D') for pair-wise linkage disequilibrium (LD) were assessed by Haploview Software [34]. *P*<0.05 was considered statistically significant. Bonferroni correction was used to adjust the significance level of a statistical test to protect against Type I errors when multiple comparisons were being made.

## Results

### D-loop Variations in Breast Cancer

**Figure 1:**
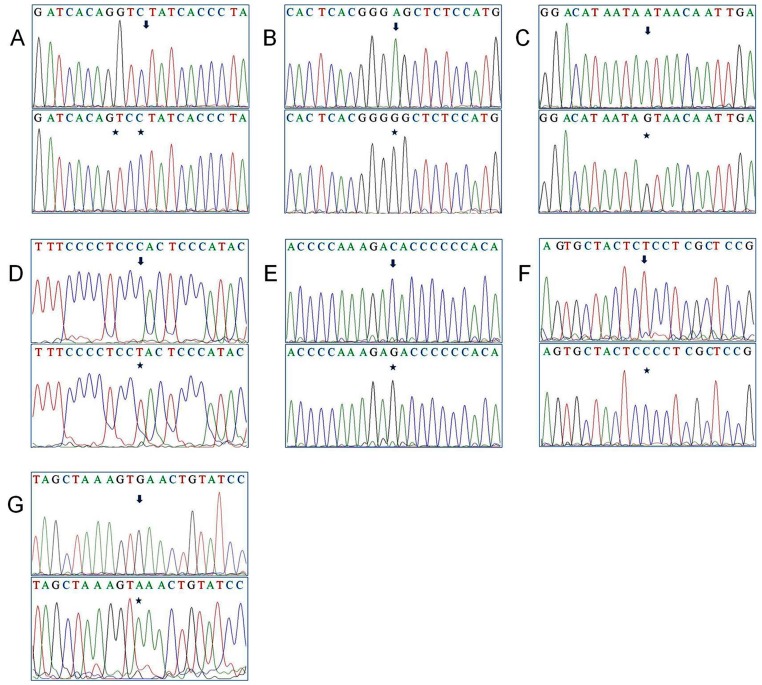
Novel mutations observed in D-loop region of breast cancer patients: (A) 12 ‘C’ insertion; (B) A37G transition; (C) A238G transition; (D) C463T transition; (E) C566G transversion; (F) T16445C transition; (G) G16485A transition.

All the subjects were successfully sequenced (n = 420). The average frequency of SNPs in breast cancer group was (1747/213 = 8.2) comparatively higher than in controls (1395/207 = 6.73). We identified 7 novel mutations in the D-loop region of breast cancer patients (Figure 1; Table 2). Among them 2 were nucleotide insertions and remaining 7 were base substitutions. Additionally, we observed 170 reported polymorphisms in the D-loop region of cases and/or controls (Table 3; Table S1). Most of them were single base substitutions (Y or R transitions). Overall, among the identified 170 reported polymorphisms 147 were base substitutions, 13 were nucleotide deletions and 10 were nucleotide insertions. Polymorphisms were predominantly located in HVR1 (60%) than in HVR2 (30%) of D-loop region. Twenty six of the 170 reported polymorphisms have >5% minor allele frequency in either patients and/or controls (Table 3). Two of these SNPs, *310‘C’* insertion (*P* = 0.018) and *T16189C* (*P* = 0.001) showed significantly elevated frequency in breast cancer patients compared to controls.

**Table 2:** Novel mutations detected in mitochondrial D-loop region of breast cancer patients.

Serial number	Nucleotide position	rCRS	Base change	IUPAC Code	Frequency
1.	12 ins C	–	C	–	1
2.	A37G	A	G	R	3
3.	A238G	A	G	R	1
4.	C463T	C	T	Y	2
5.	C566G	C	G	S	1
6.	T16445C	T	C	Y	4
7.	G16485A	G	A	R	2

rCRS: Revised Cambridge Reference Sequence.

IUPAC: International Union of Pure and Applied Chemistry.

**Table 3:** Mitochondrial D-loop polymorphisms with >5% minor allele frequency observed in breast cancer patients and/or controls.

					Frequency				
Serial number	Nucleotide position	rCRS	Base change	IUPA Code	CS	CT	*P*- value^1^	χ ^2^ value	Association
1.	G66T	G	T	K	11	8	0.521	0.41	
2.	A73G	A	G	R	168	152	0.190	1.715	
3.	A93G	A	G	R	13	8	0.292	1.107	
4.	T146C	T	C	Y	31	42	0.120	2.405	
5.	T152C	T	C	Y	39	32	0.435	0.607	
6.	T195C	T	C	Y	23	16	0.278	1.174	
7.	T195A	T	A	W	13	18	0.309	1.032	
8.	A263G	A	G	R	176	181	0.167	1.905	
9.	T310 ins C	T	C ins		75	51	**0.018**	5.589	
10.	C316 ins C	C	C ins		141	128	0.351	0.867	
11.	T489C	T	C	Y	83	67	0.158	1.992	
12.	CA522-3 del	CA	–		41	33	0.373	0.791	
13.	C525 del	C	–		17	9	0.122	2.386	
14.	A16051G	A	G	R	25	21	0.601	0.273	
15.	G16129A	G	A	R	30	19	0.117	2.452	
16.	T16172C	T	C	Y	11	8	0.521	0.41	
17.	T16189C	T	C	Y	38	16	**0.001**	9.579	Type 2 Diabetes; Cardiomyopathy
18.	C16223T	C	T	Y	97	98	0.711	0.137	Endo carci
19.	G16274A	G	A	R	27	21	0.415	0.664	
20.	C16278T	C	T	Y	11	9	0.694	0.154	
21.	T16304C	T	C	Y	16	12	0.481	0.496	
22.	T16311C	T	C	Y	31	22	0.225	1.467	
23.	A16318T	A	T	W	16	11	0.358	0.843	
24.	G16319A	G	A	R	15	11	0.462	0.54	
25.	T16362C	T	C	Y	23	27	0.477	0.505	
26.	T16519C	T	C	Y	143	122	0.081	3.031	

1 Fisher’s exact test (2×2 table at 1 df); *P*<0.05.

rCRS: Revised Cambridge Reference Sequence; CS: Cases; CT: Controls.

IUPAC: International Union of Pure and Applied Chemistry.

**Figure 2:**
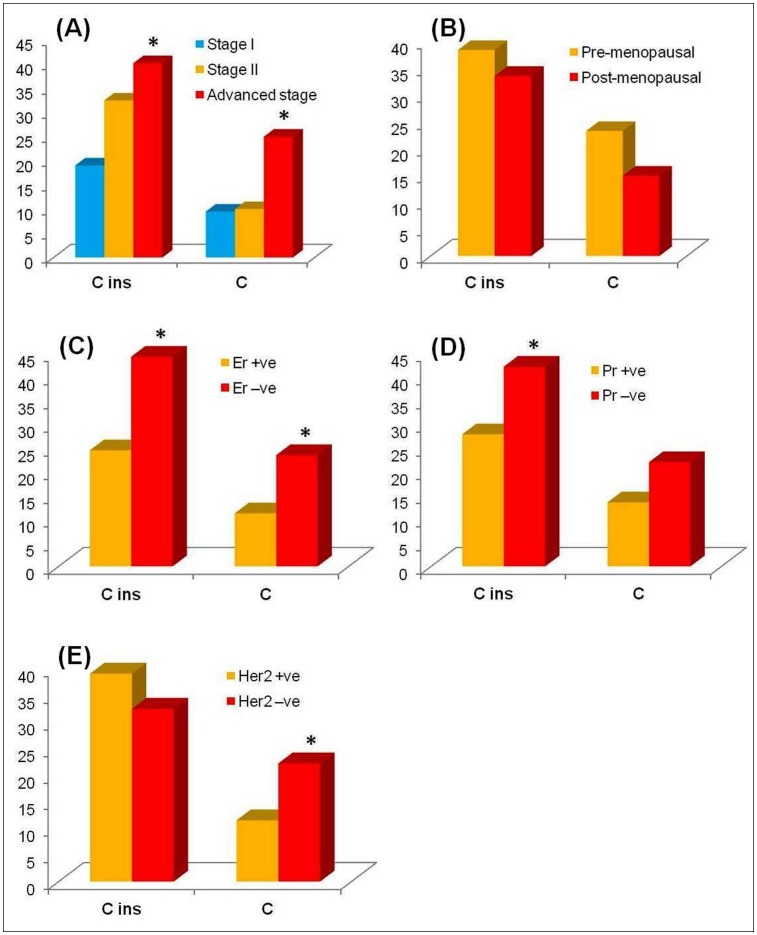
Graphical representation of minor allele frequencies of significant D-loop SNPs in breast cancer patients with different clinical parameters: (A) breast cancer stage; (B) menopausal status; (C) estrogen receptor status; (D) progesterone receptor status; (E) human epidermal growth factor receptor 2 status. Asterisk (*) indicates the significant difference (*P*<0.05, as determined by the Student’s t-test) between patient groups with different clinical parameters. Percentage values were used for statistical analysis.

Genotype frequencies of significant D-loop SNPs were further analysed based on the clinical parameters of breast cancer patients (Figure 2). The *310‘C’* insertion showed significantly increased frequency in Er –ve, Pr –ve and advanced stage breast cancer patients. For the *T16189C* SNP, significantly increased *‘C’* allele frequency was observed in Er –ve, Her2–ve and advanced stage breast cancer patients.

### Haplotype Analysis

**Figure 3:**
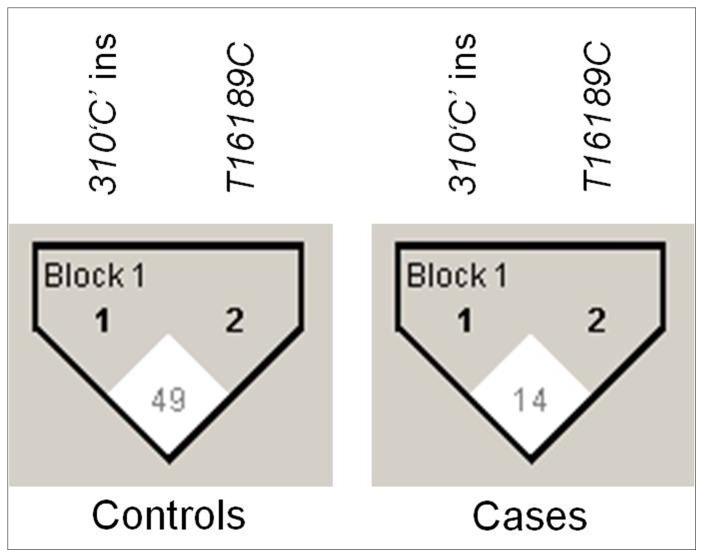
Linkage disequilibrium (LD) analysis of significant D-loop SNPs in cases and controls: Haploview plots are presented along the single nucleotide polymorphisms studied. The pair-wise linkage disequilibrium values (D′ = 0–100) of all significant SNPs are given in each diamond. A value of 100 represents maximum possible linkage disequilibrium.

To analyze the combined effect of significant D-loop SNPs on breast cancer risk, the haplotype frequencies for significant loci (*310‘C’* insertion and *T16189C*) and the standardized disequilibrium coefficient (D^'^) for pair-wise linkage disequilibrium (LD) were estimated (Table 4; Figure 3). Our results showed different pattern of LD in patients and controls. Particularly, the *310‘C’* insertion and *T16189C* showed strong LD in controls (D^'^ = 0.49) compared to patients (D^'^ = 0.14).

**Table 4:** Haplotype frequencies of significant D-loop polymorphisms observed in breast cancer patients and controls.

Haplotypes		Haplotype frequency					
310	16189	Cases	Controls	*P* value^1^	χ ^2^ value	Odds ratio	95% CI
T	T	117	142			Reference	
T	C	21	14	0.09904	2.721	1.8099	0.8933 to 3.6668
C ins	T	58	49	0.11567	2.475	1.4347	0.9145 to 2.2508
C ins	C	17	2	0.00019	13.914	5.8586	2.3101 to 14.8581

1 Fisher’s exact test (2×2 table at 1 df) *P*<0.0125 (after Bonferroni correction).

CI: Confidence Interval.

Our haplotype analysis indicates the *310T/16189T* as most common haplotype in south Indian women. Hence, relative risk of each haplotype was calculated by using this as reference. Bonferroni correction was used to adjust the significance level of a statistical test to protect against Type I errors. Since we have 4 haplotypes, the Bonferroni correction should be 0.05/4 = 0.0125. Therefore, a *P*-value less than 0.0125 was considered significant. Our results indicate that the *310‘C’ ins/16189C* (*P* = 0.00019) haplotype significantly increases breast cancer risk while the remaining haplotypes were not indicative for the disease risk.

## Discussion

In the present study, we investigated the prevalence of mitochondrial D-loop variations in breast cancer patients. Recent investigations revealed a key role of ROS induced oxidative stress in breast carcinogenesis [10]. Mitochondria are major source for cellular ROS generation [16]–[17]. Mitochondrial D-loop is a hot spot for mtDNA alterations and is important for regulation of mitochondrial genome replication and expression [22]. Hence, D-loop variations may lead to alterations in ETC there by enhances cellular ROS production. This directed our attention on this mtDNA region. Previously, some investigators have reported somatic/noninheritable D-loop variants in breast cancer [27]. However, the strong familial tendency of the disease suggests a possible inheritable genetic susceptibility. The inheritance pattern of the mitochondrial genome (maternal) leads to the gradual accumulation of mutations in successive generations. Hence, the altered mitochondrial alleles may act as inheritable predisposing factors for several diseases. In addition to the germline/congenital mutations that may predispose to breast cancer, somatic mutations may arise during the cancer development which may be cause or consequence of the disease. Therefore, in this study we mainly focused on the identification of germline D-loop variants in the breast cancer patients.

D-loop polymorphisms have been extensively investigated in various human diseases [23]–[26]. Few scientific groups have also reported association of D-loop polymorphisms with breast cancer risk in different ethnic groups including Indians [28]–[30]; however, none of the investigators have reported entire D-loop variations in breast cancer patients of Indian origin. In this study, for the first time, we reported the entire D-loop variations in breast cancer patients of south Indian origin. SNPs appear to be common in population with an average of 6 to 8 per each individual in reference to rCRS. The actual number of SNPs may be less if the reference sequence was of Indian origin. When compared with control, higher frequencies of SNPs in breast cancer patients indicate that a high SNP frequency in D-loop seems to result in predisposition to breast cancer. Furthermore, our results showed a significant association between D-loop SNPs at np 310 (*‘C’* insertion), 16189 (*T/C*) and breast cancer risk. Interestingly, these two significant SNPs are located in microsatellite loci of mitochondrial genome.

The *310‘C’* insertion, the most common microsatellite instability (MSI) of mitochondrial genome, has been associated with various multifactorial disorders [25], [35]. The np 310 was located within a homopolymeric C-stretch between np 303–315 interrupted by thymine (HVR II: np 57-333) and was reported to be a mutational hotspot [36]. Moreover, it is the replication primer binding site of mtDNA and is located in the ‘conserved sequence block II’ (CSB II) of heavy strand which contributes to the formation of a persistent RNA-DNA hybrid to initiate the mtDNA replication [37]. The RNA-DNA hybrid formation is dependent on this GC-rich element and the efficiency of hybrid formation is influenced by sequences 5′ to the hybrid, including the CSB II element [38]. In addition, exact CSB II sequence is crucial for proper mtDNA transcription [39]. Premature transcriptional termination or reduced transcription occurs if particular MSI arise in np 282-300 or 304-300 of the mtDNA sequences respectively, whereas complete transcriptional termination occurs in the 289–319 mutants [39]. Thus alterations in this repeat could lead to functional impairment of mitochondria and may result in a pro-tumorigenic phenotype of the carrier. Furthermore, elevation of *310‘C’* insertion frequency in the advanced stage, Er –ve and Pr –ve groups supports the significance of this mitochondrial variant in conferring breast cancer risk. Some of the earlier studies have reported association between *315‘C’* insertion and breast cancer risk (instead of *310‘C’* insertion) [30]. This discrepancy could be due to the genetic and ethnic variability among populations studied.

The *T16189C* is another strong mutational hotspot of mitochondrial D-loop [38] and has been associated with several multifactorial disorders [26], [40]. It generates an uninterrupted poly-C tract (np 16180–16195) in the D-loop region and may also lead to heteroplasmic length variation of the poly-C tract (>10 cytosines) in different mtDNA molecules of a single person [41]. Mitochondrial single-strand DNA-binding protein (mtSSB) bound efficiently to interrupted poly-C compared to the uninterrupted poly-C variant [26]. In addition, np 16184–16193 region was located in the 7S DNA binding site which is crucial for the regulation of mtDNA synthesis [42]. Thus, uninterrupted poly-C variant might reduce mtDNA replication and content. Reduced mtDNA content could affect the efficiency of the ETC, lower the ATP:ADP ratio, and enhance the ROS generation [18]. Increased ROS and decreased ATP:ADP ratio could contribute to the onset of several multifactorial diseases [35], [43]. In our study, the uninterrupted poly-C variant showed significantly high frequencies in breast cancer patients compared to controls (*P* = 0.0019). Moreover, the frequency of the *‘C’* allele is also elevated in breast cancer patients with advanced stage, Er –ve and Her2–ve status. Since Er –ve and Her2–ve statuses are considered to develop in to a more aggressive form of cancer, the *16189C* allele might also be associated with increased metastasis in the patients.

Furthermore, the *310‘C’ ins/16189C* (*P* = 0.00019) haplotype may have increased risk for breast cancer. Interestingly, the *310‘C’ins/16189C* haplotype has mutated alleles at both of the loci. Although it is a rare haplotype, its relative frequency was significantly higher in cases than in controls (Table 4). The mutant alleles may alter the replication and/or transcription of mitochondrial genome which may lead to enhanced ROS generation. Increased ROS generation has been reported in several human diseases including cancer [18]. Susceptibility to estrogen-inducible diseases like breast cancer is known to be influenced by mitochondrial dysfunction, because the normal metabolism of estradiol through redox-cycling intermediates may also generate local ROS and oxidative damage that facilitates neoplastic transformation [44]. ROS has been shown to be involved in aggressive growth and metastatic spread of breast cancer cells [11]. Higher endogenous oxidative stress and ROS detoxification pathways have been reported in breast cancer cells [10], [12]. All these observations strongly link the mitochondrial dysfunction with the breast cancer risk.

In conclusion, mitochondrial D-loop alterations may constitute inherent risk factors for breast cancer risk in south Indian population. The analysis of D-loop alterations might help to identify patients at high risk for disease outcome. To the best of our knowledge, this is the first report demonstrating the correlation between entire D-loop alterations and breast cancer risk in Indians. However our findings should be validated in independent cohort studies with large sample size to reduce the chance of confounding factors affecting study outcome.

## Supporting Information

Table S1 **Mitochondrial D-loop polymorphisms with <5% minor allele frequency observed in breast cancer patients and/or controls.**
(DOC)
