# Supplementary material for: Mitochondrial Control Region Alterations and Breast Cancer Risk: A Study in South Indian Population
Source: PLoS One. 2014 Jan 30;9(1):e85363. doi: 10.1371/journal.pone.0085363 (PMC3907410; doi:10.1371/journal.pone.0085363)
Supplement: Table S1 — Mitochondrial D-loop polymorphisms with <5% minor allele frequency observed in breast cancer patients and/or controls. (DOC) [file pone.0085363.s001.doc]

**Table S1.**

Mitochondrial D-loop polymorphisms with < 5% minor allele frequency observed in breast cancer patients and/or controls

|  |  |  |  |  | **Frequency** | |
| --- | --- | --- | --- | --- | --- | --- |
| **S.No** | **Nucleotide**  **position** | **rCRS** | **Base change** | **IUPAC**  **Code** | **CS** | **CT** |
|  | G9 del | G | - |  | 1 | 0 |
|  | C41T | C | T | Y | 1 | 0 |
|  | T63 del | T | - |  | 2 | 0 |
|  | C64T | C | T | Y | 1 | 1 |
|  | 66 ins T | - | T |  | 7 | 2 |
|  | A95C | A | C | M | 1 | 0 |
|  | T139C | T | C | Y | 2 | 0 |
|  | G143A | G | A | R | 1 | 0 |
|  | 150 ins T | - | T |  | 6 | 4 |
|  | C150T | C | T | Y | 6 | 3 |
|  | C151 del | C | - |  | 3 | 4 |
|  | T152 del | T | - |  | 9 | 5 |
|  | 152 ins C | - | C |  | 4 | 5 |
|  | A153G | A | G | R | 4 | **1** |
|  | C182T | C | T | Y | 3 | 0 |
|  | A189G | A | G | R | 5 | 3 |
|  | 193 ins T | - | T |  | 3 | 1 |
|  | C194T | C | T | Y | 2 | 4 |
|  | T196 del | T | - |  | 3 | 1 |
|  | C198T | C | T | Y | 2 | 0 |
|  | T199C | T | C | Y | 7 | 4 |
|  | A200G | A | G | R | 4 | 2 |
|  | T204C | T | C | Y | 1 | 6 |
|  | G207A | G | A | R | 6 | 3 |
|  | A214G | A | G | R | 1 | 0 |
|  | A215G | A | G | R | 2 | 0 |
|  | T217C | T | C | Y | 4 | 3 |
|  | G228A | G | A | R | 3 | 3 |
|  | A234G | A | G | R | 5 | 0 |
|  | T239C | T | C | Y | 3 | 0 |
|  | A240G | A | G | R | 3 | 1 |
|  | A241G | A | G | R | 2 | 0 |
|  | T246C | T | C | Y | 5 | 7 |
|  | A249 del | A | - |  | 3 | 1 |
|  | C268T | C | T | Y | 2 | 0 |
|  | C271T | C | T | Y | 1 | 1 |
|  | T279C | T | C | Y | 1 | 1 |
|  | C295T | C | T | Y | 4 | 3 |
|  | A297G | A | G | R | 1 | 0 |
|  | T310C | T | C | Y | 7 | 1 |
|  | 315 ins C | - | C |  | 2 | 0 |
|  | G316C | G | C | S | 1 | 1 |
|  | T318C | T | C | Y | 1 | 0 |
|  | T334C | T | C | Y | 1 | 0 |
|  | A373G | A | G | R | 1 | 2 |
|  | A385G | A | G | R | 3 | 0 |
|  | C447G | C | G | S | 5 | 3 |
|  | C456T | C | T | Y | 3 | 1 |
|  | C461T | C | T | Y | 4 | 7 |
|  | C462T | C | T | Y | 2 | 3 |
|  | T466C | T | C | Y | 1 | 0 |
|  | T482C | T | C | Y | 7 | 3 |
|  | T485C | T | C | Y | 4 | 0 |
|  | G499A | G | A | R | 1 | 1 |
|  | C511T | C | T | Y | 3 | 1 |
|  | G513A | G | A | R | 3 | 0 |
|  | C514A | C | A | M | 3 | 0 |
|  | A523C | A | C | M | 1 | 0 |
|  | G545C | G | C | S | 1 | 0 |
|  | C548T | C | T | Y | 1 | 0 |
|  | C568T | C | T | Y | 1 | 1 |
|  | 574 ins C | - | C |  | 2 | 0 |
|  | A574C | A | C | M | 2 | 2 |
|  | C575T | C | T | Y | 1 | 0 |
|  | C16069T | C | T | Y | 7 | 3 |
|  | T16086C | T | C | Y | 2 | 3 |
|  | T16092C | T | C | Y | 5 | 5 |
|  | T16093C | T | C | Y | 7 | 4 |
|  | C16111T | C | T | Y | 2 | 3 |
|  | T16124C | T | C | Y | 1 | 1 |
|  | T16126C | T | C | Y | 9 | 6 |
|  | G16145A | G | A | R | 5 | 5 |
|  | T16154C | T | C | Y | 4 | 3 |
|  | A16163G | A | G | R | 1 | 1 |
|  | C16167T | C | T | Y | 1 | 1 |
|  | C16169T | C | T | Y | 2 | 0 |
|  | 16169 ins C | - | C |  | 2 | 0 |
|  | C16179 del | C | - |  | 1 | 1 |
|  | C16179T | C | T | Y | 4 | 5 |
|  | A16182C | A | C | M | 3 | 5 |
|  | A16183 del | A | - |  | 8 | 3 |
|  | C16184T | C | T | Y | 5 | 0 |
|  | C16188T | C | T | Y | 2 | 5 |
|  | 16193 ins C | - | C |  | 3 | 0 |
|  | C16193T | C | T | Y | 1 | 2 |
|  | A16206C | A | C | M | 3 | 1 |
|  | A16207G | A | G | R | 3 | 2 |
|  | T16209C | T | C | Y | 5 | 5 |
|  | G16213A | G | A | R | 3 | 0 |
|  | C16214T | C | T | Y | 2 | 0 |
|  | C16214A | C | A | M | 1 | 10 |
|  | A16215G | A | G | R | 1 | 1 |
|  | C16218T | C | T | Y | 1 | 0 |
|  | C16222T | C | T | Y | 2 | 0 |
|  | C16223 del | C | - |  | 1 | 0 |
|  | A16227G | A | G | R | 1 | 2 |
|  | A16230G | A | G | R | 3 | 1 |
|  | T16231C | T | C | Y | 5 | 8 |
|  | C16234T | C | T | Y | 6 | 4 |
|  | C16239T | C | T | Y | 5 | 4 |
|  | C16242T | C | T | Y | 2 | 1 |
|  | T16243C | T | C | Y | 2 | 0 |
|  | C16245T | C | T | Y | 1 | 2 |
|  | A16254G | A | G | R | 1 | 1 |
|  | C16256G | C | G | S | 1 | 0 |
|  | C16256T | C | T | Y | 1 | 2 |
|  | C16260T | C | T | Y | 3 | 3 |
|  | C16261T | C | T | Y | 1 | 6 |
|  | C16266T | C | T | Y | 7 | 9 |
|  | C16270T | C | T | Y | 4 | 2 |
|  | C16286T | C | T | Y | 2 | 0 |
|  | C16290T | C | T | Y | 1 | 0 |
|  | C16291T | C | T | Y | 7 | 1 |
|  | C16292T | C | T | Y | 5 | 3 |
|  | A16293C | A | C | M | 1 | 1 |
|  | C16294T | C | T | Y | 2 | 1 |
|  | C16295G | C | G | S | 1 | 0 |
|  | C16295T | C | T | Y | 1 | 1 |
|  | C16296T | C | T | Y | 3 | 0 |
|  | T16297C | T | C | Y | 1 | 0 |
|  | G16303A | G | A | R | 1 | 0 |
|  | A16309G | A | G | R | 4 | 3 |
|  | A16318C | A | C | M | 1 | 1 |
|  | C16320T | C | T | Y | 6 | 0 |
|  | T16324C | T | C | Y | 2 | 0 |
|  | T16325C | T | C | Y | 1 | 1 |
|  | G16346A | G | A | R | 1 | 0 |
|  | T16352C | T | C | Y | 4 | 2 |
|  | C16354T | C | T | Y | 2 | 0 |
|  | C16355 del | C | - |  | 2 | 4 |
|  | T16356C | T | C | Y | 3 | 4 |
|  | T16357C | T | C | Y | 1 | 1 |
|  | T16368C | T | C | Y | 4 | 1 |
|  | G16390A | G | A | R | 2 | 2 |
|  | G16398A | G | A | R | 5 | 0 |
|  | A16399T | A | T | W | 6 | 1 |
|  | C16400T | C | T | Y | 1 | 0 |
|  | G16438A | G | A | R | 2 | 0 |
|  | A16463G | A | G | R | 1 | 1 |
|  | A16482G | A | G | R | 2 | 0 |
|  | A16497G | A | G | R | 2 | 1 |
|  | A16524G | A | G | R | 1 | 2 |
|  | G16526 del | G | - |  | 4 | 0 |
|  | C16527T | C | T | Y | 5 | 3 |

**rCRS**: Revised Cambridge Reference Sequence; **CS:** Cases; **CT:** controls
